# Green coffee methanolic extract and silymarin protect against CCl4-induced hepatotoxicity in albino male rats

**DOI:** 10.1186/s12906-020-03186-x

**Published:** 2021-01-07

**Authors:** Haddad A. El Rabey, Samar M. Rezk, Mohamed I. Sakran, Ghena M. Mohammed, Omar Bahattab, Maha J. Balgoon, Mohamed A. Elbakry, Nadia Bakry

**Affiliations:** 1 Biochemistry Department, Faculty of Science, University of Tabuk, Tabuk, Saudi Arabia; 2 Bioinformatics Department, Genetic Engineering and Biotechnology Research Institute, University of Sadat City, Sadat City, Egypt; 3 Clinical Nutrition Department, Mahalla Hepatology Teaching Hospital, Gharbyia, El-Mahalla El-Kubra, Egypt; 4 Biochemistry section, Chemistry Department, Faculty of Science, Tanta University, Tanta, Egypt; 5 Department of Nutrition and Food Science, Faculty of Home Economics, University of Tabuk, Tabuk, Saudi Arabia; 6 Biology Department, Faculty of Science, University of Tabuk, Tabuk, Saudi Arabia; 7 Biochemistry Department, Faculty of Science, King Abdulaziz University, Jeddah, Saudi Arabia; 8 Bone Marrow Transplantation and Cord Blood Unit, Mansoura University Children Hospital, Mansoura, Egypt

**Keywords:** Antioxidants, Green coffee, Hepatotoxicity, Irisin, Oxidative stress

## Abstract

**Background:**

During the last few decades, patients worldwide have been interested in using alternative medicine in treating diseases to avoid the increased side effects of chemical medications. Green coffee is unroasted coffee seeds that have higher amounts of chlorogenic acid compared to roasted coffee. Green coffee was successfully used to protect against obesity, Alzheimer disease, high blood pressure and bacterial infection.

**Methods:**

This study aimed to investigate the probable protective activity of the green coffee methanolic extract, silymarin and their combination on CCl_4_-induced liver toxicity in male rats. Thirty Sprague – Dawley male albino rats were divided into 5 groups; control negative (G1) just got the vehicle (olive oil) and the other four groups received CCl_4_ dissolved in olive oil through an intraperitoneal injection and were divided into untreated control positive group (G2), the third group (G3) was treated with green coffee methanolic extract, the fourth group (G4) was treated with silymarin, and the fifth group (G5) was treated with a combination of green coffee methanolic extract and silymarin.

**Results:**

In the positive control group treated with CCl_4_ (G2), the CCl_4_-induced toxicity increased lipid peroxidation, IL-6, kidney function parameters, liver function enzymes, total cholesterol, triglycerides and low-density lipoproteins, and decreased irisin, antioxidants, CYP450 and high-density lipoprotein levels. Hepatic tissues were also injured. However, treating the injured rats in G3, G4 and G5 significantly improved the altered parameters and hepatic tissues.

**Conclusions:**

Green coffee methanolic extract, silymarin, and their combination succeeded in protecting the male rats against CCl4 hepatotoxicity due to their antioxidant activity. Effect of green coffee methanolic extract mixed with silymarin in G5 was more efficient than that of green coffee methanolic extract in G3 or silymarin in G4.

**Supplementary Information:**

The online version contains supplementary material available at 10.1186/s12906-020-03186-x.

## Background

The liver, which is considered the biggest metabolic organ in the body, plays an essential role in modifying and clearing synthetic chemicals which can lead to the impairment of its function that can be destroyed by the overdoses of certain drugs or sometimes when introduced within the recommended therapeutic doses [1]. Liver diseases are mostly caused by infections, autoimmune disorders, excessive alcohol drinking and toxic chemicals that may harm liver cells principally by instigating oxidative damages or lipid peroxidation [2]. Hepatotoxicity is mainly caused as a result of oxidative stress resulting from free radicals produced by numerous biochemical procedures that damage proteins, DNA, lipids and proteins, causing major diseases like rheumatoid, cancer, degenerative aging processes, cardiovascular disease and arthritis [3].

Carbon tetrachloride (CCl_4_) is broadly used to induce liver damage in animal models for experimental purposes, since it is assorted as a hepatotoxin [4–7]. This chemical was successfully applied to rats [5–8], mice [9], and birds [10]. Induction of oxidative stress is one of the main mechanisms underlying CCl_4_ hepatotoxicity [5]. Thus, antioxidants supplementation could be helpful in the treatment of its hepatotoxic effects.

The main function of antioxidants is to counteract oxidative injury brought by free radicals by interfering with the oxidation procedure through radical searching and chelating metal particles [4]. Many natural products have been assigned as antioxidants and they showed protective effects against CCl_4_-induced hepatotoxicity, e.g. *Lycium barbarum* in mice [5], olive oil and *Nigella sativa* oil in male rats [6] and *Moringa oleifera* in rats [7]. In this context, the flavonoid silymarin is broadly used in cases of hepatic diseases because of its significant antioxidant and hepatoprotective activities [11, 12]. Its supplementation attenuated the hepatocyte functional and histopathological alterations in acetaminophen-induced normotensive and hypertensive rats [13]. Also, Baradaran et al. [10] stated that silymarin has a potential to alleviate CCl_4_-induced hepatotoxicity by up-regulating CAT, GPx, and Mn-SOD hepatic genes in CCl_4_-challenged birds.

Coffee is another natural product with powerful antioxidant activity [11, 12]. Billions of coffee cups are consumed in the world every year [14]. Despite the invigorating impact of caffeine (which represents the main component of coffee) on the risk of developing acute coronary syndromes [15], spontaneous abortion [16] and type 1 diabetes mellitus in children [17], it has antioxidant activities and a positive protective effect against the risk of metabolic syndrome [18]. For this reason, most studies on coffee were focused on the beneficial protective activity of caffeine [19, 20], because of its antioxidant properties and diminishing mortality chances against different degenerative diseases such as malignant growth, diabetes, liver diseases and Parkinson’s disease [21, 22]. Also, caffeine has advances in ingesting glucose utilization in serum epinephrine [23]. Moreover, the pre-practice uses of caffeine improve oxidative stress, lipolysis and ventilation [24, 25]. As the positive effect of coffee consumption on human health is attributed to the potent antioxidant activity of its polyphenolic content, the roasting of coffee may alter the polyphenolic profile of the beans and consequently affect its antioxidant activity [26]. Also, the progressive antioxidant activity and polyphenols content are decreased with roasting, whereas light and medium roasted coffees might protect cells from oxidative stress damages [27]. Green coffee, which contains chlorogenic acids (caffeic and ferulic acid) [25], is therefore of particular interest. In vitro trials showed that green coffee extract exhibited antidiabetic activities including inhibition of adipogenesis [28].

Recently, Abdelaal et al. [29] compared the hepatoprotective effect of silymarin and green coffee extract against thioacetamide-induced hepatotoxicity on the histological and immunohistochemical levels in rats. However, the therapeutic efficiency of these two natural antioxidants, whether supplemented alone or in combination, against CCl_4_-induced liver oxidative damage in rats has not been previously evaluated.

The current study aimed to study the protective activity of the green coffee methanolic extract, silymarin and their combination against CCl_4_ induced hepatotoxicity. To this end, lipid peroxidation, antioxidant enzymes, liver function, kidney function, cytochrome P450, cholesterol, triacylglycerol, low-density lipoprotein, and irisin as markers for CCl_4_ hepatic toxicity in male rats were estimated.

## Methods

### Chemicals

The chemicals used in this study were of molecular biology and diagnostic grade. They were purchased from Sigma (St. Louis, MO, USA). Pure virgin olive oil was purchased from a local market. Silymarin was purchased from SEDICO Company, Egypt.

### Green coffee methanolic extract preparation

Dried seeds of green Brazilian coffee were purchased from a local market. The green coffee seeds were identified by Prof. Haddad El Rabey (Botanist). A specimen was deposited at the herbarium of Faculty of Science, Botany Department, Mansoura University, Egypt (Accession No. #FSH00134–2019).

The seeds were milled to powder using a mill. A 200 g of the green coffee powder was added to 1 liter methanol (90%), agitated on a shaker at room temperature for 5 days. The methanol was completely evaporated using a rotary evaporator attached to a vacuum pump. For the 20% methanolic extract preparation, 20 g of the extract was mixed with 2 ml of tween 80 (suspending agent), and then the mixture was suspended in 100 ml of distilled water according to the method described by Adebayo et al. [30].

### Animals and experimental design

A total of 30 adult Sprague–Dawley strain albino male rats (weighing 159.86 ± 6.96 g) were purchased from the Agricultural Research Center, Giza, Egypt. The rats were housed in polypropylene cages (6/cage). Before the beginning of the experiment, the animals were kept under observation (to be sure that they are healthy), and subjected to acclimatization for the laboratory environment during 1 week. Room temperature and humidity were adjusted at 23 °C and 60%, respectively. Also, the light cycle was fixed at 12 h. The guidelines of the National Institutes of Health (NIH) for the care and use of laboratory animals (NIH Publication, Number 85–23, Revised 1985) were applied to all animal experiments of this study. Also, this study was ethically approved by The Institutional Research Board (IRB) Committee of Mansoura Faculty of Medicine, Mansoura University, Egypt. During the acclimatization period and the whole experiment, food and water were given ad libitum. The acclimatized rats were divided into five groups as follows: negative control group (G1) that received only the vehicle (olive oil), and the other four rat groups received a single dose of CCl4 (1 ml/kg bw) dissolved in olive oil (1:1 volume) in the 1st and 4th day every week of the experiment through an intraperitoneal (IP) injection [6] then they were divided into four groups as follows: untreated positive control group (G2), and three treated groups (G3, G4 and G5) with IP. The third group (G3) was treated with a daily dose of 5 ml/kg of 20% green coffee methanolic extract [7] using oral gavage, the fourth group (G4) was treated with 0.05 g/kg of silymarin [7] and the fifth group (G5) was treated with 0.05 g/kg of silymarin and 5 ml/kg of 20% green coffee methanolic extract, respectively for 30 days. All animal experiments were approved by The Ethical Committee of Mansoura University.

### Blood collection

The rats were euthanatized by 2.5% halothane (1:20 halothane vapor: air ratio, flow rate was 70%V/min) at the end of the experiment and blood samples were collected, centrifuged for 5 min at 3000 rpm, and then the serum was transferred into clean tubes for biochemical analysis.

### Liver homogenate preparation

The liver was dissected out, washed with saline solution, divided into two parts, the first part was fixed in 10% formalin for the histological examination and the second part was homogenized in ice-cold phosphate buffer (pH 7.4), and then centrifuged at 4000 rpm for 15 min. Lipid peroxidation (MDA) as a marker of oxidative stress and antioxidant biomarkers were determined in the supernatant.

### Determination of biochemical parameters

The activity of all liver function enzymes was estimated in serum using Sigma Aldrich commercial kits from Sigma Aldrich Company (USA). Alanine aminotransferase (ALT) and aspartate aminotransferase (AST) activities were estimated as described by Reichling and Kaplan [31], while alkaline phosphatase activity was estimated as described by Mossner et al. [32]. Total cholesterol (TC), Triglycerides (TAG), high-density lipoprotein (HDL), and low density lipoprotein (LDL) levels were measured according to the manufacturer protocols of bioMerieux kit (France). Serum total protein, bilirubin and albumin were determined according to the protocol of Diamond (Germany). Serum urea, uric acid and creatinine were estimated as described by Kanter [33], Fossati et al. [34], and Bonsens and Taussky [35], respectively using a Human kit (Germany).

Irisin levels were determined in serum using Cono Biotech Co. Ltd. kit (China), interleukin-6 (IL-6) was determined using MyBiosource Kit (San Diego, USA), whereas cytochrome P450 (CYP 450) was determined using Abbexa kit from Cambridge (UK), according to the instructions of the suppliers.

Liver homogenates were used for the determination of catalase (CAT), superoxide dismutase (SOD), and glutathione transferase (GST) activities, as well as total antioxidant capacity (TAC), and reduced glutathione (GSH) and malondialdehyde (MDA) levels using Biodiagnostic Kit (Egypt), according to the method described by the supplier.

### Histological study

The liver tissue was immediately fixed in 10% formalin, dehydrated in ethanol (70, 80 and 90%), cleared in xylene, embedded in paraffin, and then sectioned by a microtome. The sections were then stained with hematoxylin and eosin (H&E) as described by Bancroft and Stevens [36].

### Statistical analysis

The obtained data were expressed as means ± standard deviation and they were analyzed by one-way analysis of variance (ANOVA statistical measure). Duncan’s test was used as a post hoc test for comparison of significance between groups using the statistical package program (SPSS) version 17.0 [37]. The SigmaPlot software (AUTOSIGNAL V1.7) was used in plotting graphics.

## Results

### Biochemical analysis

Table 1 showed that serum AST, ALT, and ALP activities, as well as bilirubin, creatinine, uric acid, urea, cholesterol, triacylglycerol, and low-density lipoprotein levels were significantly increased, whereas albumin, total protein and high-density lipoproteins levels were significantly decreased in the positive control group (G2) compared to the negative control group (G1). However these parameters were partially restored to their normal values approaching the normal values in the treated groups (G3, 4 and 5) compared to G2. The combination of green coffee methanolic extract and silymarin in G5 was more efficient in reducing the activities of all liver function enzymes compared to the other groups (G3 and G4).

**Table 1:** Effect of green coffee, silymarin and their combination on ALT, AST, ALP), total protein (TP), albumin, bilirubin, creatinine, uric acid (UA), blood urea nitrogen (BUN) and lipids in CCl_4_ induced hepatotoxic rats

	ALP (U/L)	AST (Ul)	ALT (U/l)	Cholesterol (mg/dl)	TG (mg/dl)	HDL (mg/dl)	LDL (mg/dl)	BUN (mg/dl)	UA (mg/dl)	Creatinine (mg/dl)	Bilirubin (mg/dl)	TP (g/dl)	Albumin (g/dl)	LDH (U/L)
**Negative control group** **(G1)**	164.0 ± 8.62^#^	22.33 ± 2.8^#^	21.66 ± 3.55^#^	163.5 ± 6.89^#^	134.0 ± 11.34^#^	90.36 ± 6.44^#^	46.33 ± 1.63^#^	22.0 ± 2.6^#^	4.25 ± 0.18^#^	0.71 ± 0.15^#^	0.42 ± 0.03^#^	6.5 ± 0.14^#^	5.43 ± 0.28^#^	175.16 ± 12.54^#^
**Positive control group** **(Group 2)**	283.33 ± 6.94*	91.83 ± 5.49*	98.5 ± 5.2*	277.33 ± 7.11*	250.83 ± 8.28*	18.66 ± 1.75*	208.0 ± 7.72*	64.0 ± 3.22*	7.28 ± 0.29*	2.51 ± 0.28*	1.51 ± 0.19*	3.5 ± 0.28*	2.31 ± 0.2*	446.0 ± 8.94*
**Green coffee methanolic extract** **(Group 3)**	254.33 ± 5.35*^#^	76.33 ± 2.16*^#^	84.66 ± 3.14*^#^	258.5 ± 5.46*^#^	222.66 ± 3.72*^#^	32.0 ± 1.54*^#^	183.0 ± 6.44*^#^	56.83 ± 1.94*^#^	6.86 ± 0.1*^#^	1.93 ± 0.1*^#^	0.89 ± 0.04*^#^	4.38 ± 0.2*^#^	2.96 ± 0.15*^#^	404.0 ± 8.89*^#^
**Silymarin** **(Group 4)**	190.0 ± 4.89*^#^	53.33 ± 4.22*^#^	54.83 ± 3.43*^#^	223.33 ± 4.92*^#^	197.16 ± 5.49*^#^	34.5 ± 2.16*^#^	149.16 ± 4.99*^#^	46.0 ± 2.09*^#^	5.98 ± 0.16*^#^	1.3 ± 0.14*^#^	0.67 ± 0.01*^#^	5.08 ± 0.14*	4.1 ± 0.12*^#^	360.5 ± 5.78*^#^
**Combination of green coffee methanolic extract and silymarin** **(Group 5)**	164.16 ± 5.52^#^	40.33 ± 1.21*^#^	45.0 ± 2.0*^#^	204.33 ± 7.68*^#^	178.5 ± 6.7*^#^	40.33 ± 1.21*^#^	127.83 ± 6.3*^#^	35.83 ± 2.22*^#^	5.23 ± 0.12*^#^	0.855 ± 0.018^#^	0.54 ± 0.025^#^	5.95 ± 0.23*^#^	4.78 ± 0.17*^#^	214.16 ± 7.25*^#^
**Negative control group** **(G1)**	164.0 ± 8.62^#^	22.33 ± 2.8^#^	21.66 ± 3.55^#^	163.5 ± 6.89^#^	134.0 ± 11.34^#^	90.36 ± 6.44^#^	46.33 ± 1.63^#^	22.0 ± 2.6^#^	4.25 ± 0.18^#^	0.71 ± 0.65^#^	0.42 ± 0.03^#^	6.5 ± 0.14^#^	5.43 ± 0.28^#^	175.16 ± 12.54^#^
**Positive control group** **(Group 2)**	283.33 ± 6.94*	91.83 ± 5.49*	98.5 ± 5.2*	277.33 ± 7.11*	250.83 ± 8.28*	18.66 ± 1.75*	208.0 ± 7.72*	64.0 ± 3.22*	7.28 ± 0.29*	2.51 ± 0.28*	1.51 ± 0.19*	3.5 ± 0.28*	2.31 ± 0.2*	446.0 ± 8.94*
**Green coffee methanolic extract** **(Group 3)**	254.33 ± 5.35*^#^	76.33 ± 2.16*^#^	84.66 ± 3.14*^#^	258.5 ± 5.46*^#^	222.66 ± 3.72*^#^	32.0 ± 1.54*^#^	183.0 ± 6.44*^#^	56.83 ± 1.94*^#^	6.86 ± 0.1*^#^	1.93 ± 0.1*^#^	0.89 ± 0.04*^#^	4.38 ± 0.2*^#^	2.96 ± 0.15*^#^	404.0 ± 8.89*^#^
**Silymarin** **(Group 4)**	190.0 ± 4.89*^#^	53.33 ± 4.22*^#^	54.83 ± 3.43*^#^	223.33 ± 4.92*^#^	197.16 ± 5.49*^#^	34.5 ± 2.16*^#^	149.16 ± 4.99*^#^	46.0 ± 2.09*^#^	5.98 ± 0.16*^#^	1.3 ± 0.14*^#^	0.67 ± 0.01*^#^	5.08 ± 0.14*	4.1 ± 0.12*^#^	360.5 ± 5.78*^#^
**Combination of green coffee methanolic extract and silymarin** **(Group 5)**	164.16 ± 5.52^#^	40.33 ± 1.21*^#^	45.0 ± 2.0*^#^	204.33 ± 7.68*^#^	178.5 ± 6.7*^#^	40.33 ± 1.21*^#^	127.83 ± 6.3*^#^	35.83 ± 2.22*^#^	5.23 ± 0.12*^#^	0.855 ± 0.018^#^	0.54 ± 0.025^#^	5.95 ± 0.23*^#^	4.78 ± 0.17*^#^	214.16 ± 7.25*^#^
**Negative control group** **(G1)**	164.0 ± 8.62^#^	22.33 ± 2.8^#^	21.66 ± 3.55^#^	163.5 ± 6.89^#^	134.0 ± 11.34^#^	90.36 ± 6.44^#^	46.33 ± 1.63^#^	22.0 ± 2.6^#^	4.25 ± 0.18^#^	0.71 ± 0.65^#^	0.42 ± 0.03^#^	6.5 ± 0.14^#^	5.43 ± 0.28^#^	175.16 ± 12.54^#^
**Positive control group** **(Group 2)**	283.33 ± 6.94*	91.83 ± 5.49*	98.5 ± 5.2*	277.33 ± 7.11*	250.83 ± 8.28*	18.66 ± 1.75*	208.0 ± 7.72*	64.0 ± 3.22*	7.28 ± 0.29*	2.51 ± 0.28*	1.51 ± 0.19*	3.5 ± 0.28*	2.31 ± 0.2*	446.0 ± 8.94*
**Green coffee methanolic extract** **(Group 3)**	254.33 ± 5.35*^#^	76.33 ± 2.16*^#^	84.66 ± 3.14*^#^	258.5 ± 5.46*^#^	222.66 ± 3.72*^#^	32.0 ± 1.54*^#^	183.0 ± 6.44*^#^	56.83 ± 1.94*^#^	6.86 ± 0.1*^#^	1.93 ± 0.1*^#^	0.89 ± 0.04*^#^	4.38 ± 0.2*^#^	2.96 ± 0.15*^#^	404.0 ± 8.89*^#^
**Silymarin** **(Group 4)**	190.0 ± 4.89*^#^	53.33 ± 4.22*^#^	54.83 ± 3.43*^#^	223.33 ± 4.92*^#^	197.16 ± 5.49*^#^	34.5 ± 2.16*^#^	149.16 ± 4.99*^#^	46.0 ± 2.09*^#^	5.98 ± 0.16*^#^	1.3 ± 0.14*^#^	0.67 ± 0.01*^#^	5.08 ± 0.14*	4.1 ± 0.12*^#^	360.5 ± 5.78*^#^
**Combination of green coffee methanolic extract and silymarin** **(Group 5)**	164.16 ± 5.52^#^	40.33 ± 1.21*^#^	45.0 ± 2.0*^#^	204.33 ± 7.68*^#^	178.5 ± 6.7*^#^	40.33 ± 1.21*^#^	127.83 ± 6.3*^#^	35.83 ± 2.22*^#^	5.23 ± 0.12*^#^	0.855 ± 0.018^#^	0.54 ± 0.025^#^	5.95 ± 0.23*^#^	4.78 ± 0.17*^#^	214.16 ± 7.25*^#^
**Negative control group** **(G1)**	164.0 ± 8.62^#^	22.33 ± 2.8^#^	21.66 ± 3.55^#^	163.5 ± 6.89^#^	134.0 ± 11.34^#^	90.36 ± 6.44^#^	46.33 ± 1.63^#^	22.0 ± 2.6^#^	4.25 ± 0.18^#^	0.71 ± 0.65^#^	0.42 ± 0.03^#^	6.5 ± 0.14^#^	5.43 ± 0.28^#^	175.16 ± 12.54^#^
**Positive control group** **(Group 2)**	283.33 ± 6.94*	91.83 ± 5.49*	98.5 ± 5.2*	277.33 ± 7.11*	250.83 ± 8.28*	18.66 ± 1.75*	208.0 ± 7.72*	64.0 ± 3.22*	7.28 ± 0.29*	2.51 ± 0.28*	1.51 ± 0.19*	3.5 ± 0.28*	2.31 ± 0.2*	446.0 ± 8.94*
**Green coffee methanolic extract** **(Group 3)**	254.33 ± 5.35*^#^	76.33 ± 2.16*^#^	84.66 ± 3.14*^#^	258.5 ± 5.46*^#^	222.66 ± 3.72*^#^	32.0 ± 1.54*^#^	183.0 ± 6.44*^#^	56.83 ± 1.94*^#^	6.86 ± 0.1*^#^	1.93 ± 0.1*^#^	0.89 ± 0.04*^#^	4.38 ± 0.2*^#^	2.96 ± 0.15*^#^	404.0 ± 8.89*^#^
**Silymarin** **(Group 4)**	190.0 ± 4.89*^#^	53.33 ± 4.22*^#^	54.83 ± 3.43*^#^	223.33 ± 4.92*^#^	197.16 ± 5.49*^#^	34.5 ± 2.16*^#^	149.16 ± 4.99*^#^	46.0 ± 2.09*^#^	5.98 ± 0.16*^#^	1.3 ± 0.14*^#^	0.67 ± 0.01*^#^	5.08 ± 0.14*	4.1 ± 0.12*^#^	360.5 ± 5.78*^#^
**Combination of green coffee methanolic extract and silymarin** **(Group 5)**	164.16 ± 5.52^#^	40.33 ± 1.21*^#^	45.0 ± 2.0*^#^	204.33 ± 7.68*^#^	178.5 ± 6.7*^#^	40.33 ± 1.21*^#^	127.83 ± 6.3*^#^	35.83 ± 2.22*^#^	5.23 ± 0.12*^#^	0.855 ± 0.018^#^	0.54 ± 0.025^#^	5.95 ± 0.23*^#^	4.78 ± 0.17*^#^	214.16 ± 7.25*^#^

The results are expressed as the M ± SD. * shows a statistically significant difference (*P* < 0.05) and ^#^ significant at *p* < 0.05 compared with the positive control group (G2)

**Fig. 1:**
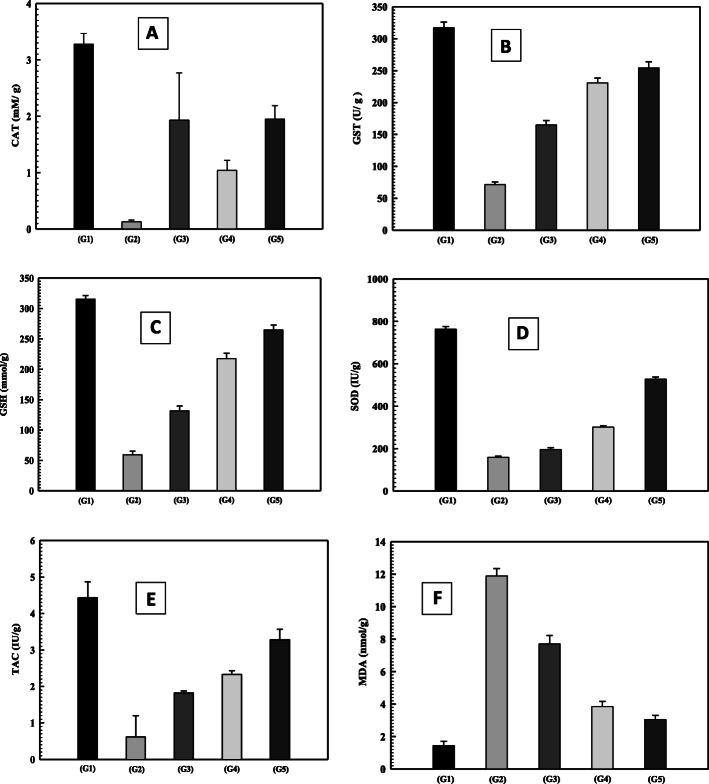
Effect of green coffee, silymarin and their combination on antioxidants and lipid peroxidation in CCl_4_ induced hepatotoxic rats. **a:** catalase (CAT), **b**: glutathione transferase (GST),**c**: glutathione reduced (GSH), **d**:superoxide dismutase (SOD), **e**:total antioxidant capacity (TAC), **f**:malondialdehyde (MDA) G1: negative control group, G2: untreated positive control group, G3 treated with green coffee methanolic extract, G4 treated with silymarin and G5 treated with silymarin and green coffee methanolic extract

Figure 1(a,b,c,d,e), (and supplementary Table 1) showed that CCl4 toxicity in the positive control group (G2) significantly decreased the activities of catalase (CAT), superoxide dismutase (SOD) and glutathione transferase (GST), total antioxidant capacity (TAC), and the level of reduced glutathione (GSH) in the liver tissue homogenate. However, these markers were increased in rats treated with green coffee methanolic extract, silymarin and their synergic combination (G1, G2 and G3, respectively) compared to the positive control group in G2

.

Figure 1f (and supplementary Table 1) also showed that; CCl_4_ toxicity in the positive control group (G2) significantly elevated the amount of lipid peroxidation (MDA) when compared to the negative control in G1. Whereas treating with green coffee methanolic extract, silymarin and their combination in G3, G4 and G5, respectively significantly reduced this high peroxidation level, although still significantly higher than that of the untreated negative control group (G1). Also, the combination of green coffee methanolic extract and silymarin in G5 was more efficient in reducing lipid peroxidation and increasing antioxidants compared to other groups (G3 and G4).

**Fig. 2:**
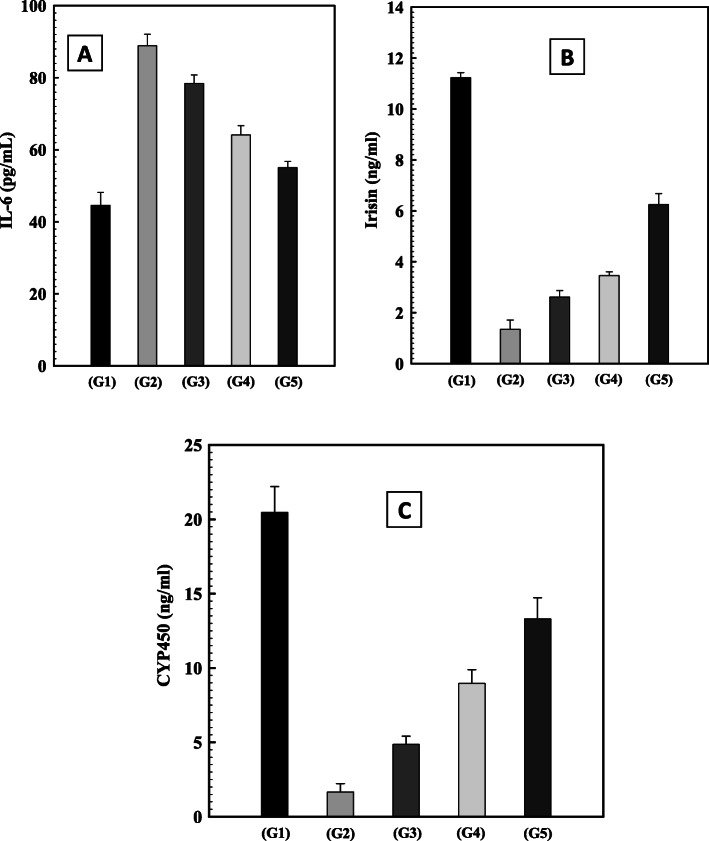
Effect of green coffee methanolic extract, silymarin and their combination onIL-6, Irisin and P450 levels in CCl_4_induced hepatotoxic male rats. **a**: Interleukin-6 (IL-6), **b**: Irisin,**c**:CYP450. G1: negative control group, G2: untreated positive control group, G3 treated with green coffee methanolic extract, G4 treated with silymarin and G5 treated with silymarin and green coffee methanolic extract

Figure 2 (a,b,c) (and supplementary Table 2) showed similar findings in G2. A significant decrease in the levels of both irisin and CYP450 (*p* < 0.05) and an increase in IL-6 level (*P* < 0.05) in comparison to the negative control group (G1) was observed in G2. Rats treated with green coffee methanolic extract, silymarin and their combination (G3, G4 and G5, respectively) showed a significant increase in both irisin and CYP450 levels (*p* < 0.05), in comparison with those in G2. Moreover, treating with the mixture of green coffee-methanolic extract and silymarin in G5 was more efficient in increasing both irisin and CYP450 and decreasing IL-6

Furthermore, the possible correlations among the studied parameters were evaluated (See supplementary Table 3). A positive correlation was observed between antioxidants and each other. A positive correlation was also observed between antioxidants, CYP 450 and irisin. In contrast, a significant negative correlation was found between MDA and antioxidants. Furthermore, a positive and highly significant correlation was observed between CYP 450 and irisin. Also, a highly negative correlation was observed between IL-6 and both CYP 450 and irisin (Supplementary Table 3).

### Histopathology

**Fig. 3:**
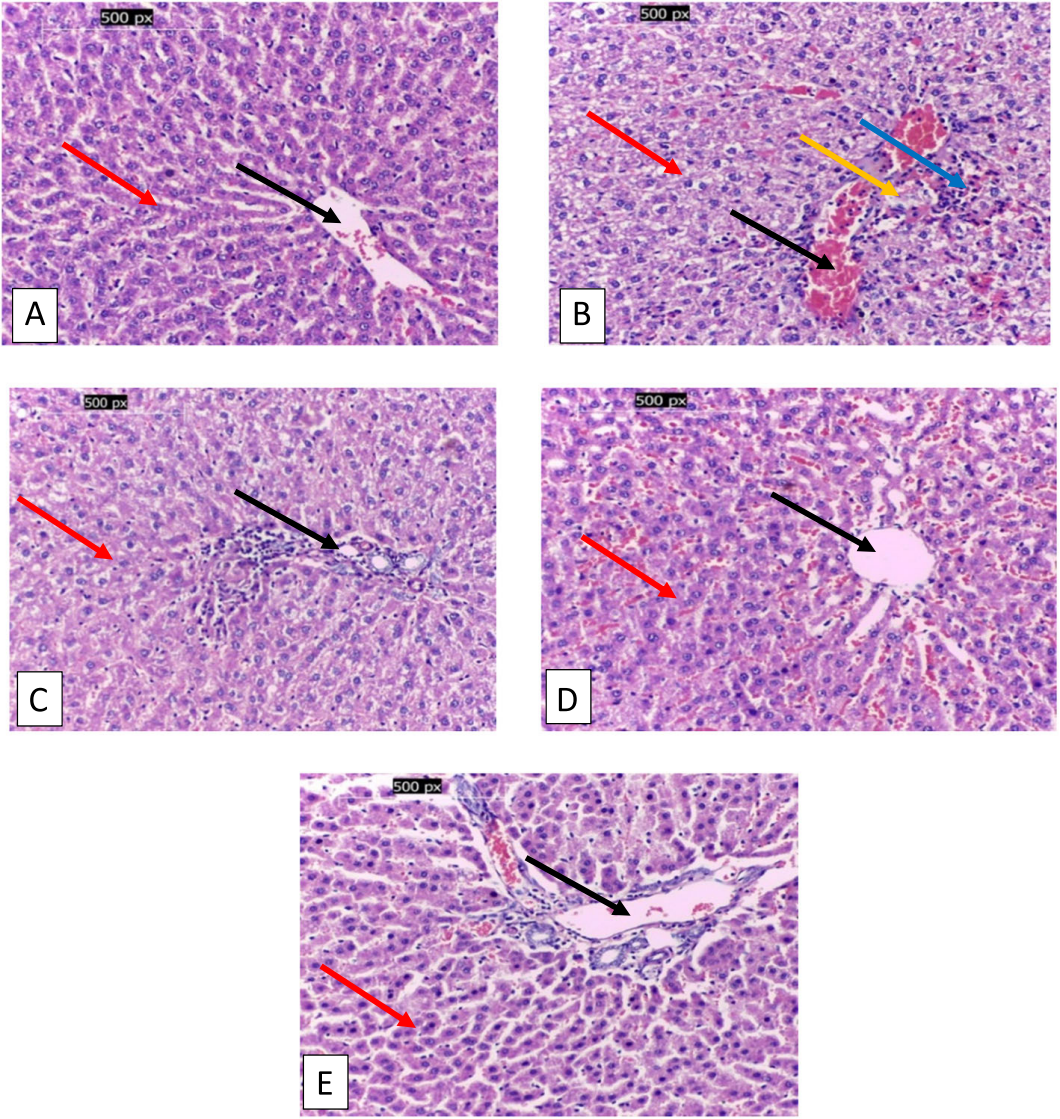
**a,b,c,d,e**. Microscopic images of liver tissue in the groups under study. **a**: Control negative group, showing the normal histological structure of hepatic tissue with normal cells (red arrow) and vein (black arrow), **b**: Control positive group, showing fatty infiltrated and degenerated hepatocyte (red arrow), congested vein and sinusoids (black arrow), degenerated biliary duct (orange arrow) and bridging necrotic cells (blue arrow), **c**: Green coffee methanolic extract group, showing nearly normal hepatocytes (red arrow) with mildly inflammated vein (black arrow). **d**: Silymarin group, showing mild fatty degeneration, mild inflammation and interstitial edema (red arrow) with normal vein (black arrow). **e**: Combination of green coffee methanolic extract and silymarin group, showing nearly normal hepatocytes (red arrow) and vein (black arrow). (X 400, H&E stains)

Figure 3 (a,b,c,d,e) showed the histopathological examinations of rats under study. Figure 3a showed the normal hepatic tissues of the untreated negative control group (G1). The CCl_4_ toxicity (Fig. 3b) severely damaged the liver tissue in G2. The hepatic tissues of G2 showed fatty infiltrated and degenerated hepatocytes, congested hepatic vein, and sinusoid degenerated biliary duct and bridging necrotic cells. However, rats treated with green coffee methanolic extract, silymarin and their combination in G3 (Fig. 3c), G4 (Fig. 3d) and G5 (Fig. 3e), respectively showed ameliorated hepatic tissues, particularly in G5 that showed nearly normal hepatocytes without any congestion

## Discussion

The present results demonstrated that green coffee methanolic extract has a great antioxidant activity that ameliorated all the altered biochemical parameters and nearly restored the damaged hepatic tissues to their normal aspect. It is also known that green coffee decreases blood lipid levels, enhances energy metabolism and expenditure, supports weight management and enhances glucose tolerance [38]. Therefore, our idea was to study the probable protective effect of green coffee methanolic extract, silymarin and their combination against the CCl_4_-induced hepatotoxicity.

Injecting the animals with CCl_4_ for hepatotoxicity induction in G2, caused a critical increment of alkaline phosphatase (ALP) and transaminases (AST and ALT) activities reflecting the functional status of the liver and showing hepatocytes damage [6, 7, 39]. The histopathology of the liver of the CCl_4_-induced hepatic toxicity group (G2) showed injured hepatic tissues that led to the significant elevation of these liver function parameters in serum. Also, the total protein (TP) level was reduced in the CCl_4_ rats showing the pulverization in the quantity of hepatic cells, which may result in a diminished hepatic ability to synthesize proteins [6, 7, 29]. Whereas the administration of the green coffee methanolic extract, silymarin, or their combination significantly and advantageously improved serum levels of these liver function parameters approaching them to the negative control group levels. Mixing the green coffee methanolic extract with silymarin was more efficient in protecting against the CCl_4_-induced hepatic toxicity than silymarin or the coffee extract in G3 and G4, respectively.

Similarly, induction of hepatotoxicity in G2 caused a critical increment in total cholesterol, triglycerides and LDL levels and a significant decrease in HDL levels in the liver tissue homogenate. The disturbance in the metabolism of phospholipids and protein synthesis may be engaged with irregular lipoprotein levels [8]. In the current study, the green coffee methanolic extract accompanied by silymarin significantly increased HDL levels and decreased levels of cholesterol, TG and LDL [29]. This result was in agreement with that of Yukawa et al. [40] who stated that consumption of 24 g coffee/day, for 1 week, reduces serum cholesterol and low-density lipoproteins, while it was not in accordance with that of McAnlis et al. [41],who reported that coffee does not affect serum lipids. Other beneficial effects of chlorogenic acid (a bioactive constituent of green coffee) on the improvement of lipid profile and insulin resistance (IR) were investigated in experimental and human studies [42]. Sudeep et al. [43] stated that administration of chlorogenic acid also reduces plasma levels of cholesterol, free fatty acids and TG in hyperlipidemic Wistar rats.

In the present study, CCl_4_-induced hepatic toxicity increased the inflammation and the oxidative stress as revealed by the increase in malondialdehyde (MDA) and IL-6 levels. This result is in agreement with that of Li et al. [44], Al-Seeni et al. [6] and Elbakry et al. [7]. The overproduction of free radicals increased the level of lipid peroxidation followed by the formation of adducts with nucleic acids and cellular proteins that eventually diminished the function of hepatocytes [9]. However, the consumption of green coffee methanolic extract and silymarin reduced the oxidative stress and adjusted the antioxidant redox system and the pro-inflammatory chemokines and cytokines (IL-6). Our results showed that consumption of green coffee methanolic extract accompanied by silymarin caused a suppressive effect on oxidative stress and confirmed authors’ previous reports concerning the antioxidant activity of green coffee and its ability to trap hydroxyl radicals and superoxide anions [45, 46]. Our results also showed that the antioxidative effect of green coffee methanolic extract accompanied by silymarin on enzymatic antioxidant systems was reflected by increased concentrations of antioxidants (TAC, GSH, GST, CAT and SOD) and a decreased lipid peroxidation that may be ascribed to its richness in caffeine, which is considered as an important component of coffee. Moreover, the antioxidant activity of green coffee makes it able to trap hydroxyl radicals or superoxide anions because of its content of both phenolic compounds and chlorogenic acid [45]. Moreover, it is known that the reactive oxygen species (ROS) are harmful when present at high concentrations, however a certain level of ROS is important in utilizing redox cell signaling to sustain cellular homeostasis [46].

One interesting observation in this study is that the consumption of green coffee methanol extract escorted by silymarin had a protective effect on oxidative stress (MDA(and antioxidant redox system. Also, our results showed that consumption of green coffee methanolic extract accompanied by silymarin causes a suppressive effect on oxidative stress and confirmed the findings of other authors showing the antioxidant power of green coffee [21, 22, 29]. Furthermore, oxidative DNA damage was decreased; and glutathione level and glutathione reductase activities were increased after consumption of green coffee [29, 46].

Decreased CYPP450 enzymatic activity in the positive control group as a result of CCl_4_ toxicity increased the generation of reactive metabolites and oxidative stress, ultimately leading to increased toxicity [6, 7]. Meanwhile, the levels of CYP450 and irisin were improved in green coffee methanolic extract with silymarin group due to the antioxidant activity of green coffee that ameliorated both oxidative stress and lipid peroxidation, suggesting the positive effect of green coffee in attaining normal hepatic function since irisin is correlated with hepatic lipid metabolism [8].

The green coffee bean extract contains many polyphenols, especially chlorogenic acid (CGA), which have antioxidant, anti-inflammatory and antimutagenic activities [47]. It also contains bioactive compounds such as caffeine, theophylline, theobromine, cafestol, kahweol, tocopherols and trigonelline [6], hydroxycinnamic acids such as caffeic and ferulic acids and quinic acid esters called chlorogenic acids (CGAs) [48].

It is also worth mentioning that our study is a novel one concentrating on testing the protective activity of the green coffee methanolic extract, silymarin and their combination on oxidative stress. Abdelaal et al. [29] studied the effect of silymarin and green coffee on the histological and immunohistochemical changes resulting from thioacetamide-induced liver toxicity. In our study, we measured highly specific biochemical parameters, such as lipid peroxidation, IL-6, kidney function parameters, liver function enzymes, total cholesterol, triglycerides and low-density lipoproteins, irisin, antioxidants, CYP450 and high-density lipoprotein levels to evaluate liver CCl_4_-induced toxicity and its alleviation after green coffee and silymarin supplementation.

### Limitation of the study

More studies are needed to determine the mechanism(s) of the hepatoprotective effect of green coffee methanolic extract and its bioactive compounds. More than one mechanism underlying this effect may be involved because of the mixture of bioactive components present in green coffee.

## Conclusions

CCl_4_ toxicity in rats promotes the pro-inflammatory cytokines as IL-6 to be emitted at the site of aggravation because of liver damage and produce a lot of reactive oxygen species (ROS) prompting oxidative stress. However, green coffee methanolic extract and silymarin have been demonstrated to have hepatoprotective activity in the exposed rats. These beneficial effects of green coffee methanolic extract may be due to its possible ability to improve irisin, CYP450, and antioxidant properties that restored liver function, oxidative stress biomarkers and inflammatory cytokine nearly to their normal values. Also, administration of green coffee methanolic extract accompanied with silymarin in G5 was more efficient in protecting against CCl_4_-induced hepatic toxicity than either green coffee methanolic extract in G3 or silymarin in G4.

## Supplementary Information

**Additional file 1: Supplementary Table 1.** Effect of green coffee, ilymarin and their combination on CAT, GST, GSH, SOD, TAC, MDA in CCl_4_ induced hepatotoxic rats.

**Additional file 2: Supplementary Table 2.** Effect of green coffee, silymarin and their combination on IL-6, irisin and P450 levels in CCl_4_ induced hepatotoxic rats.

**Additional file 3: Supplementary Table 3.** Correlations coefficient (r) values in some measured parameters in all groups

## Data Availability

The data of this study are available with the corresponding author upon request.

## Abbreviations

CAT: Catalase
CCl4: Carbon tetrachloride
G1: The first rats group, just got the vehicle (olive oil)
G2: The second rats group (positive controls) injected with CCl_4_
G3: Rats group injected with CCl_4_ and treated with a daily dose of 20% (w/w) green coffee methanolic extract.
G4: The fourth rats group injected with CCl_4_ and treated with 0.05 g/kg silymarin
G5: The fifth rats group injected with CCl_4_ and treated with a combination of green coffee methanolic extract and silymarin as in G3 and G4
GSH: Reduced glutathione.
GST: Glutathione-S-transferase.
ROS: Reactive oxygen species.
TAC: Total antioxidant capacity
TG: Triglycerides
TP: Total protein
P450: Cytochrome P450
IL-6: Interleukin-6
