# Supplementary material for: Green coffee methanolic extract and silymarin protect against CCl4-induced hepatotoxicity in albino male rats
Source: BMC Complement Med Ther. 2021 Jan 7;21:19. doi: 10.1186/s12906-020-03186-x (PMC7792057; doi:10.1186/s12906-020-03186-x)
Supplement: Supplementary file 1 — Additional file 1: Supplementary Table 1. Effect of green coffee, ilymarin and their combination on CAT, GST, GSH, SOD, TAC, MDA in CCl4 induced hepatotoxic rats. [file 12906_2020_3186_MOESM1_ESM.docx]

**Supplementary Table 1. Effect of green coffee, silymarin and their combination on CAT, GST, GSH, SOD, TAC, MDA in CCl_4_ induced hepatotoxic rats.**

|  | **CAT**  **(mM/ g)** | **GST**  **(U/ g )** | **GSH**  **(mmol/g)** | **SOD**  **(IU/g)** | **TAC**  **(IU/g)** | **MDA**  **(nmol/ g)** |
| --- | --- | --- | --- | --- | --- | --- |
| **Negative control group**  **(G1)** | 3.28 ± 0.19^#^ | 317.11 ± 8.9^#^ | 315.33 ± 6.04^#^ | 762.83 ± 12.15^#^ | 4.43 ± 0.44^#^ | 1.46 ± 0.24^#^ |
| **Positive control group**  **(G2)** | 0.13 ± 0.03* | 71.31 ± 4.22* | 59.33 ± 5.6* | 158.83 ± 5.56* | 0.616 ± 0.58* | 11.9 ± 0.45* |
| **Green coffee methanolic extract**  **(G3)** | 1.93 ±0.84 *^#^ | 165.0 ± 6.75*^#^ | 131.66 ± 7.756*^#^ | 195.66 ± 8.14*^#^ | 1.82 ±0.053*^#^ | 7.71 ± 0.51*^#^ |
| **Silymarin**  **(G4)** | 1.04 ± 0.18*^#^ | 230.83 ± 7.65*^#^ | 217.5 ± 8.57*^#^ | 301.0 ± 7.4*^#^ | 2.33 ± 0.1*^#^ | 3.85 ± 0.32*^#^ |
| **Combination of green coffee methanolic extract and silymarin**  **(G5)** | 1.95 ± 0.24*^#^ | 254.31 ± 9.52*^#^ | 264.83 ± 7.93*^#^ | 527.5 ± 9.43*^#^ | 3.28 ± 0.29*^#^ | 3.05 ± 0.26*^#^ |

The results are expressed as the M ± SD. * shows a statistically significant difference (P < 0.05) and ^#^ significant at p< 0.05 compared with the positive control (G2).
