# Supplementary material for: Green coffee methanolic extract and silymarin protect against CCl4-induced hepatotoxicity in albino male rats
Source: BMC Complement Med Ther. 2021 Jan 7;21:19. doi: 10.1186/s12906-020-03186-x (PMC7792057; doi:10.1186/s12906-020-03186-x)
Supplement: Supplementary file 2 — Additional file 2: Supplementary Table 2. Effect of green coffee, silymarin and their combination on IL-6, irisin and P450 levels in CCl4 induced hepatotoxic rats. [file 12906_2020_3186_MOESM2_ESM.docx]

**Supplementary Table 2. Effect of green coffee, silymarin and their combination on IL-6, irisin and P450 levels in CCl_4_ induced hepatotoxic rats.**

|  | **IL-6**  **pg/mL** | **Irisin**  **ng/ml** | **CYP450**  **ng/ml** |
| --- | --- | --- | --- |
| **Negative control group (G1)** | 44.58 ± 3.59# | 11.23 ± 0.2# | 20.46 ± 1.73# |
| **Positive control group (G2)** | 88.86 ± 3.21* | 1.35 ± 0.36* | 1.66 ± 0.56* |
| **Green coffee methanolic extract (G 3)** | 78.4 ± 2.38*# | 2.62 ± 0.25*# | 4.86 ± 0.56*# |
| **Silymarin (G4)** | 64.13 ± 2.58*# | 3.46 ± 0.15*# | 8.96 ± 0.92*# |
| **Combination of green coffee methanolic extract and silymarin (G5)** | 55.05 ± 1.7 *# | 6.25 ± 0.43*# | 13.3 ± 1.42*# |

The results are expressed as the M ± SD. * shows a statistically significant difference (P < 0.05) and (^#^) Significant at p< 0.05 compared with the positive control (G2).
