# Supplementary material for: Green coffee methanolic extract and silymarin protect against CCl4-induced hepatotoxicity in albino male rats
Source: BMC Complement Med Ther. 2021 Jan 7;21:19. doi: 10.1186/s12906-020-03186-x (PMC7792057; doi:10.1186/s12906-020-03186-x)
Supplement: Supplementary file 3 — Additional file 3: Supplementary Table 3. Correlations coefficient (r) values in some measured parameters in all groups [file 12906_2020_3186_MOESM3_ESM.docx]

**Supplementary Table 3. Correlations coefficient (r) values in some measured parameters in all groups**

|  | **ALT** | **AST** | **Creatinine** | **UA** | **Cholesterol** | **MDA** | **TAC** | **CAT** | **SOD** | **GSH** | **GST** | **IL-6** | **Irisin** | **CYP450** |
| --- | --- | --- | --- | --- | --- | --- | --- | --- | --- | --- | --- | --- | --- | --- |
| **ALT** | - | .989* | .945* | .973* | .980* | .953* | -.880* | -.959* | -.939* | -.981* | -.968* | .975* | -.914* | -.968* |
| **AST** | .989* | - | .954* | .972* | .976* | .985* | -.869* | -.965* | -.940* | -.986* | -.971* | .981* | -.915* | -.965* |
| **Creatinine** | .945* | .954* | -- | .927* | .927* | .968* | -.850* | -.923* | -.870* | -.969* | -.954* | .971* | -.830* | -.899* |
| **UA** | .973* | .972* | .927* | - | .979* | .910* | -.897* | -.963* | -.970* | -.960* | -.945* | .966* | -.848* | -.970* |
| **Cholesterol** | .980* | .976* | .927* | .979* | - | .921* | -.837* | -.963* | -.961* | -.967* | -.955* | .966* | -.945* | -.976* |
| **MDA** | .953* | .985* | .968* | .910* | .921* | - | -.764* | -.923* | -.840* | -.976* | -.982* | .966* | -.815* | -.897* |
| **TAC** | -.880* | -.869* | -.850* | -.897* | -.837* | -.764* | - | .852* | .966* | .955* | .843* | .957* | .956* | .914* |
| **CAT** | -.959* | -.965* | -.923* | -.963* | -.963* | -.923* | .852* | - | .946* | .961* | .955* | -.963* | .935* | .956* |
| **SOD** | -.939* | -.940* | -.870* | -.970* | -.961* | -.840* | .966* | .946* | - | .919* | .891* | -.933* | .984* | .975* |
| **GSH** | -.981* | -.986* | -.969* | -.860* | -.967* | -.976* | .955* | .961* | .919* | - | .982* | -.984* | .981* | .949* |
| **GST** | -.968* | -.971* | -.954* | -.945* | -.955* | -.982* | .843* | .955* | .891* | .982* | - | -.967* | .881* | .937* |
| **IL-6** | .975* | .981* | .971* | .966* | .966* | .957* | -.963* | -.961* | -.933* | -.984* | -.967* | - | -.907* | -.954* |
| **Irisin** | -.914* | -.915* | -.830* | -.848* | -.945* | -.815* | .956* | .935* | .984* | .981* | .881* | -.907* | - | .967* |
| **CYP450** | -.968* | -.965* | -.899* | -.970* | -.976* | -.897* | .914* | .956* | .975* | .949* | .937* | -.954* | .967* | - |

(^*^) Significant at *p*> 0.05 in each correlation.
